# Risk for invasive and borderline epithelial ovarian neoplasias following use of hormonal contraceptives: the Norwegian–Swedish Women's Lifestyle and Health Cohort Study

**DOI:** 10.1038/sj.bjc.6601715

**Published:** 2004-03-09

**Authors:** M Kumle, E Weiderpass, T Braaten, H-O Adami, E Lund

**Affiliations:** 1 Institute of Community Medicine, Faculty of Medicine, University of Tromsø, N-9037 Tromsø, Norway; 2 Department of Medical Epidemiology, Karolinska Institutet, S-17177 Stockholm, Sweden; 3 International Agency for Research on Cancer, 150 cours Albert Thomas, F-69372 Lyon cedex 08, France

**Keywords:** invasive epithelial ovarian neoplasias, borderline epithelial ovarian neoplasias, hormonal contraceptives, cohort, women, Sweden, Norway

## Abstract

The risk of ovarian epithelial neoplasia following use of hormonal contraceptives (HC) was examined in data from the Norwegian–Swedish Women's Lifestyle and Health cohort including 103 551 women aged 30–49 years in 1991–92. Follow-up through 2000 produced 214 incident cases of histologically confirmed epithelial ovarian neoplasias (135 invasive and 79 borderline cases). Using the Cox proportional hazard models, ever having used HC was associated with a decreased relative risk of epithelial ovarian cancer of 0.6 (95% CI 0.5–0.8). The effect of duration of HC use was convincing (*P* for trend <0.0001), and more important than age at start of use or time since first or last use. There was no significant difference between the effects of combined oral contraceptives and progestins-only contraceptives on risk (*P*=0.98). Similarly, there was no significant difference between the effects of ever use of HC on invasive and borderline ovarian neoplasia (*P*=0.37). In this cohort, use of HC seems to reduce the risk of epithelial ovarian neoplasia markedly and persistently in relation to the duration of use.

That use of hormonal contraceptives (HC) may reduce the incidence of ovarian cancer is well established from both case–control (Weiss *et al*, 1981; Centers for Disease Control Cancer and Steroid Hormone Study, 1987; The WHO Collaborative Study of Neoplasia and Steroid Contraceptives, 1989; Franceschi *et al*, 1991; Whittemore *et al*, 1992; Siskind *et al*, 2000) and cohort studies (Ramcharan *et al*, 1980; Willett *et al*, 1981; Vessey *et al*, 1987; Beral *et al*, 1988, 1999; Hankinson *et al*, 1995; Vessey and Painter, 1995), as recently reviewed (IARC Working Group, 1999; La Vecchia and Franceschi, 1999). Indeed, this benefit may be strong enough to reduce population-based incidence rates in places where HC have been used extensively (Adami *et al*, 1990; Parazzini *et al*, 2000; Walker *et al*, 2002). Nevertheless, few studies have distinguished between the two subtypes of epithelial ovarian neoplasias (EON): invasive epithelial ovarian neoplasia (IEON) and borderline epithelial ovarian neoplasia (BEON). Little is known about the possible benefit of continued HC use for 10 or more years, and it is unclear for how long the effect lasts following cessation of treatment. A detailed understanding of the potential benefits of using HC may be of considerable importance both for individual women and for the public health. We present here results from a large, population-based, prospective study: the Norwegian–Swedish Women's Lifestyle and Health cohort – with detailed assessment of HC use and complete follow-up.

## MATERIAL AND METHODS

### Study cohort

In 1991–92, the Norwegian–Swedish Women's Lifestyle and Health cohort was established in Norway and Sweden. Using the unique person number, 196 000 women aged 30–49 years were sampled at random among all women in their age group from the Central Population Register in Norway and the Swedish Central Population Registry at Statistics Sweden (Lunde *et al*, 1980). In Norway, women aged 34–49 years (born 1943–57) at the time of invitation were selected from the entire country. The Swedish women were aged 30–49 years (born 1943–60) and residing in Uppsala Health Care Region. The study population is described elsewhere (Kumle *et al*, 2002). The 106 841 women who responded to the four-page self-instructed questionnaire were included in the cohort, giving a response rate of 54.5%. The questionnaire contained a set of identical questions in the two countries, including detailed assessment of contraceptive use and reproductive history together with other lifestyle habits. To facilitate recall, a colour brochure with pictures of almost all contraceptive pill packages ever sold in Norway and Sweden was included with the letter of invitation and questionnaire.

From the initial cohort, we excluded five women due to lack of vital status information (alive, dead or emigrated), 15 who were dead or had emigrated before the start of follow-up and 1681 women who reported having an invasive cancer of any type at study enrolment (information obtained from the cancer registries). In addition, 1126 women who with lacked information on use of HC and 463 who had undergone bilateral oophorectomy, and therefore not at risk for ovarian cancer, were excluded from the present analysis, leaving 103 551 women eligible for follow-up.

### Follow-up

We achieved complete follow-up through linkages between the cohort data set and various population-based registries using the individual unique national registration numbers (Lunde *et al*, 1980) assigned to all residents in Norway and Sweden. The national cancer registers, established in the 1950s in both countries, provided data on prevalent cancer cases at cohort enrolment and incident cancers diagnosed in the cohort during the follow-up. These cancer registries are estimated to be almost 100% complete (Lund, 1981; Mattsson and Wallgren, 1984), and include information on pathological tumour features.

The start of follow-up was defined as the date of return of the questionnaire. We obtained information on the dates of death for deceased persons from the death registers, and on the date of emigration from the registers of population migration. The follow-up ended on 31 December 2000, at emigration, death or diagnosis of histologically confirmed primary EON, whichever occurred first. Among the 106 841 women enrolled initially into the cohort, 789 emigrated and 1360 died during the period of follow-up.

### Exposure classification

The information about exposure to HC presented here is based on answers to questions on summary measures as ever having used, current use, total duration of use, age at first use, and use before first full-term pregnancy. We also collected information about each specific period of use. A period was defined as use of a specified HC brand for at least 1 month. Up to 10 different periods of use were reported. For each period, we asked for the age at starting use, duration of use and brand name. We calculated the time since last use as the interval between the end of use and start of the follow-up. ‘Current use’ was defined as self-reported use of HC at the time of study enrolment, or use within 1 year – regardless of how many months – before the start of follow-up. Time since first use was defined as the interval between the start of HC use and the start of follow-up. Information about commercial names or brands in each period of use made it possible to classify HC as combined oral contraceptives (COCs) and progestins-only contraceptives (POPs; pills, injectable depot medroxy-progesterone acetate or levonorgestrel implants).

We considered the use of postmenopausal hormone therapy (ever/never use) among the very few women who had used these at enrolment as either combined estrogen–progestin or estrogen only. At the start of follow-up, only women who reported a natural menopause were considered as postmenopausal (since women with a bilateral oophorectomy were excluded from all analyses). Women reporting use of postmenopausal hormone therapy before natural menopause (2902 women) and those reporting hysterectomy without bilateral oophorectomy (2541 women) were treated as having unknown menopausal status. All other women were considered as premenopausal.

### Statistical analysis

The proportional hazards assumptions were met and relative hazards were calculated using the Cox proportional hazard model (Cox, 1972) and SAS Software Package (version 8.2) considering use of HC as the independent variable and epithelial ovarian neoplasms (i.e. invasive and borderline together) and separately invasive and borderline neoplasms as dependent variables. We interpreted relative hazards as estimates of relative risk (RR), which are given with 95% confidence intervals (CI). Women who had never used HC were considered as the comparison group, if not otherwise specified.

We kept the following covariates in the final multivariate model: age at enrolment into the cohort (as a continuous variable), parity (nulliparous/parous women), use of postmenopausal hormone replacement therapy (ever/never users), menopausal status at the start of follow-up (pre/postmenopausal and unknown menopausal status), and country of residence (Sweden/Norway). Levels of recreational physical activity, educational status, age at first birth, total duration of breast-feeding, breast cancer in mother/sister, age at menarche, and body mass index (BMI, i.e., weight in kg divided by height in square-metres) were not included in the final multivariate models, because they did not improve the goodness of fit or change risk estimates meaningfully.

The characteristics of HC use such as age at first use, time since first use, time since last use, and duration of use are closely related and might therefore be confounded by each other. In the multivariate analyses of age at first use, time since first use, and time since last use, the analyses were performed both with and without duration of use as a covariate. Ordinal ranks were assigned to each category of duration of HC use when calculating the tests for linear trend. The RRs for ever use of COCs and POPs were calculated separately.

Tests for heterogeneity across subgroups of women were performed by calculating a Wald *χ*^2^ statistic for differences between log hazards. In this way, we tested the difference in risk for both invasive and borderline epithelial ovarian neoplasms with regard to never use, ever use, current use of HC, use of COCs and POPs, and duration of use of HC, COCs, and POPs.

The responsible Data Inspection Boards and Ethical Committees in both countries approved the study design, and all women provided informed consent to participate in the study.

## RESULTS

### Characteristics of the study cohort

In the study population of 103 551 women aged 30–49 years at enrolment in 1991/92, 12% were aged 30–34, 31% were aged 35–39, 29% were aged 40–44, and 28% were aged 45–49. During the follow-up through year 2000, 214 cases of primary EON were diagnosed. These were distributed as 135 cases of primary IEON and 79 cases of BEON. The median age at diagnosis was 49 years for IEON and 48 years for BEON; for both, the median year of diagnosis was 1997.

We explored the parity, menopausal status, and use of postmenopausal hormone replacement therapy as risk factors for EON, and thus as possible confounders (Table 1

**Table 1:** Characteristics of the study population at cohort enrolment, adjusted for age (The Norwegian–Swedish Women's Lifestyle and Health Cohort Study)

	**Women at risk of ovarian cancer**	**Epithelial ovarian neoplasias^a^**		**IEON**		**BEON**	
**Characteristics at cohort enrolment**	**Number (%)**	**Number (%)**	**Age-adjusted RR (95% CI)**	**Number (%)**	**Age-adjusted RR (95% CI)**	**Number (%)**	**Age-adjusted RR (95% CI)**
*Parity*							
Never parous	12 050 (11.6)	34 (15.9)	1.0 (ref)	21 (15.6)	1.0 (ref)	13 (16.5)	1.0 (ref)
Ever parous	91 501 (88.4)	180 (84.1)	0.6 (0.4–0.9)	114 (84.4)	0.6 (0.4–1.0)	66 (83.5)	0.6 (0.3–1.1)
One child	14 593 (14.1)	30 (14.0)	0.7 (0.4–1.1)	16 (11.8)	0.6 (0.3–1.1)	14 (17.7)	0.9 (0.4–1.8)
Two children	45 240 (43.7)	91 (42.5)	0.6 (0.4–0.9)	56 (41.5)	0.6 (0.4–1.0)	35 (44.3)	0.7 (0.4–1.2)
Three or more children	31 669 (30.6)	59 (27.6)	0.5 (0.4–0.8)	42 (31.1)	0.6 (0.4–1.0)	17 (21.5)	0.4 (0.2–0.9)
Per child			0.9 (0.8–1.0)		0.9 (0.8–1.0)		0.8 (0.6–0.9)

*Menopausal status at entry into the study*							
Premenopausal	94 651 (91.4)	177 (82.7)	1.0 (ref)	111 (82.2)	1.0 (ref)	66 (83.5)	1.0 (ref)
Postmenopausal	3742 (3.6)	20 (9.4)	1.8 (1.1–2.9)	15 (11.1)	1.9 (1.1–3.3)	5 (6.3)	1.5 (0.6–3.8)
Unknown	5159 (5.0)	17 (7.9)	1.2 (0.7–2.0)	9 (6.7)	0.9 (0.5–1.9)	8 (10.2)	1.8 (0.9–3.9)

*Ever used HRT*^b^							
No	99 757 (96.3)	197 (92.1)	1.0 (ref)	125 (92.6)	1.0 (ref)	72 (91.1)	1.0 (ref)
Yes	3795 (3.7)	17 (7.9)	1.5 (0.9–2.5)	10 (7.4)	1.3 (0.7–2.4)	7 (8.9)	2.0 (0.9–4.5)

a Invasive epithelial ovarian neoplasias (IEON) and borderline epithelial ovarian neoplasias (BEON) together.

b Postmenopausal hormone replacement therapy (oestrogens only, or oestrogens added to progestins).

). Parous women had a decreased risk of EOC compared to nulliparous women (RR 0.6; 95% CI 0.4–0.9). We found a slight increased risk for ovarian cancer among postmenopausal women. Use of postmenopausal hormones (HRT) before enrolment was reported by only 3.7% of the cohort, and was not associated with development of EON.

### Risk by pattern of HC use

The risk of EON was 40% lower among ever users of HC than among never users (after adjustment for age, parity, use of postmenopausal hormone replacement therapy, menopausal status, and women's country of origin). The decreased risk of EON was observed both among current and former users of HCs (Table 2

**Table 2:** Relative risks (RR) with 95% confidence intervals (CI) for ovarian epithelial neoplasias according to pattern of hormonal contraceptive use at cohort enrolment (The Norwegian–Swedish Women's Lifestyle and Health Cohort Study)

		**Epithelial ovarian neoplasias^a^**		**IEON**		**BEON**	
**Use of hormonal contraceptives at cohort enrolment**	**Women at risk of ovarian cancer**	**Number**	**RR (95% CI) Multivariate^b^**	**Number**	**RR (95% CI) Multivariate^b^**	**Number**	**RR (95% CI) Multivariate^b^**
Never users	28 019	93	1.0 (Ref.)	63	1.0 (Ref.)	30	1.0 (Ref.)
Ever users	75 533	121	0.6 (0.5–0.8)	72	0.6 (0.4–0.8)	49	0.7 (0.5–1.2)
POPs^c^	4438	6	0.5 (0.2–1.2)	2	0.3 (0.1–1.1)	4	1.0 (0.4–2.9)
COCs^d^	42 616	58	0.5 (0.4–0.7)	36	0.5 (0.3–0.8)	22	0.6 (0.3–1.0)
Both COCs and POPs or unknown brand	28 479	57	0.7 (0.5–1.0)	34	0.7 (0.4–1.0)	23	0.9 (0.5–1.5)
Current users	9805	9	0.5 (0.2–0.9)	5	0.4 (0.2–1.0)	4	0.5 (0.2–1.6)
Former users	65 728	112	0.6 (0.5–0.8)	67	0.6 (0.4–0.8)	45	0.7 (0.5–1.2)

a Invasive epithelial ovarian neoplasias (IEON) and borderline epithelial ovarian neoplasias (BEON) together.

b Adjusted for baseline information about: age (continuous variable in completed years), parity (nulliparous *vs* parous), use of postmenopausal hormone therapy (ever *vs* never), menopausal status (pre-, post-menopausal or unknown menopausal status at the start of follow-up) and country (Sweden/Norway).

c POPs=progestins-only preparations.

d COCs=Combined oral contraceptives.

). There was no significant difference between the effects of COCs and POPs on risk (*P*=0.98). Similarly, there was no significant difference in risk with regard to ever use (*P*=0.37) or current use (*P*=0.70) of HC.

Among the 2541 women reporting hysterectomy without bilateral oophorectomy at enrolment, six developed EON (four developed IEON and two developed BEON). Ever use of HC was associated with a nonsignificant 20% decrease in risk (RR 0.8; 95% CI 0.3–2.1 in an age-adjusted analysis) among these women.

### Risk by duration and time since first and last use of HCs

Risk of EON decreased with increasing duration of HC use (*P* for trend *P*<0.0001) (Table 3

**Table 3:** Relative risks (RR) with 95% confidence intervals (CI) for epithelial ovarian neoplasias according to duration of hormonal contraceptive use at cohort enrolment (The Norwegian–Swedish Women's Lifestyle and Health Cohort Study)

		**Epithelial ovarian neoplasias^a^**		**IEON**		**BEON**	
**Use of hormonal contraceptives at cohort enrolment**	**Women at risk of ovarian cancer**	**Number**	**RR (95% CI) Multivariate^b^**	**Number**	**RR (95% CI) Multivariate^b^**	**Number**	**RR (95% CI) Multivariate^b^**
Never users	28 019	93	1.0 (Ref.)	63	1.0 (Ref.)	30	1.0 (Ref.)
							
*Duration of HC use*							
<1 year	8493	21	0.9 (0.5–1.4)	19	1.2 (0.7–2.0)	2	0.2 (0.1–1.0)
1–4 years	23 688	35	0.5 (0.4–0.8)	21	0.5 (0.3–0.8)	14	0.6 (0.3–1.2)
5–9 years	20 719	33	0.6 (0.4–0.9)	19	0.6 (0.3–0.9)	14	0.7 (0.4–1.4)
10–14 years	9795	13	0.5 (0.3–1.0)	5	0.3 (0.1–0.8)	8	0.9 (0.4–2.0)
15+ years	4348	1	0.1 (0.01–0.6)	1	0.1 (0.02–0.8)	—	—
*P* for trend			*P*<0.0001		*P*<0.0001		*P*=0.2
Per year of use			0.92 (0.88–0.96)		0.89 (0.84–0.94)		0.96 (0.91–1.0)

a Invasive epithelial ovarian neoplasias (IEON) and borderline epithelial ovarian neoplasias (BEON) together.

b Adjusted for baseline information about: age (continuous variable in completed years), parity (nulliparous *vs* parous), use of postmenopausal hormone therapy (ever *vs* never), menopausal status (pre-, post-menopausal or unknown menopausal status at the start of follow-up) and country (Sweden/Norway).

) and the reduced risk seemed to continue beyond 10–14 years, with a RR of less than 0.1 after 15 or more years of use. When duration of HC use was analysed as a continuous variable, the risk was reduced by 10% per year of use (Table 3).

Table 4

**Table 4:** Relative risks (RR) with 95% confidence intervals (CI) for epithelial ovarian neoplasias according to information about age at first use, time since first use and time since last use of hormonal contraceptives at cohort enrolment (The Norwegian–Swedish Women's Lifestyle and Health Cohort Study)

		**Epithelial ovarian neoplasia^a^**			**IEON**		**BEON**	
**Use of hormonal contraceptives at cohort enrolment**	**Women at risk of ovarian cancer**	**Number**	**RR (95% CI) Multivariate^b^**	**RR (95% CI) Multivariate, adjusted for duration of use**	**Number**	**RR (95% CI) Multivariate^b^**	**Number**	**RR (95% CI) Multivariate^b^**
Never users	28 019	93	1.0 (Ref.)	1.0 (Ref.)	63	1.0 (Ref.)	30	1.0 (Ref.)

*Age at first use*								
<20 years	31 262	32	0.5 (0.3–0.8)	0.6 (0.3–1.0)	21	0.5 (0.3–1.0)	11	0.4 (0.2–0.9)
20–24 years	29 014	49	0.6 (0.4–0.8)	0.7 (0.5–1.1)	25	0.4 (0.3–0.7)	24	0.8 (0.5–1.4)
25+ years	14 576	38	0.8 (0.5–1.1)	1.0 (0.6–1.5)	25	0.7 (0.5–1.1)	13	0.8 (0.4–1.4)

*Time since first use*								
0–19 years	40 402	55	0.7 (0.5–1.0)	0.9 (0.6–1.4)	33	0.7 (0.4–1.1)	22	0.7 (0.4–1.2)
20+ years	34 450	64	0.5 (0.4–0.8)	0.7 (0.5–1.1)	38	0.5 (0.3–0.7)	26	0.7 (0.4–1.3)

*Time since last use*								
0–9 years	26 799	27	0.5 (0.3–0.7)	0.9 (0.5–1.7)	17	0.5 (0.3–0.8)	10	0.4 (0.2–0.9)
10–14 years	13 928	25	0.7 (0.4–1.1)	1.1 (0.6–1.9)	11	0.5 (0.2–0.9)	14	1.1 (0.6–2.1)
15–19 years	15 205	26	0.6 (0.45–0.9)	0.8 (0.5–1.3)	17	0.6 (0.3–1.0)	9	0.6 (0.3–1.3)
20+ years	11 085	22	0.5 (0.3–0.9)	0.6 (0.4–1.0)	17	0.6 (0.3–1.0)	5	0.4 (0.2–1.0)

a Invasive epithelial ovarian neoplasias (IEON) and borderline epithelial ovarian neoplasias (BEON) together.

b Adjusted for baseline information about: age (continuous variable in completed years), parity (nulliparous *vs* parous), use of postmenopausal hormone therapy (ever *vs* never), menopausal status (pre-, post- or unknown menopausal status at the start of follow-up) and country (Sweden/Norway).

shows the RR estimates according to age at first use, time since first use, and time since last use of HC. The multivariate RR estimates are given both with and without adjustment for duration of HC use. The reduced risk associated with HC use was observed regardless of age at first use, and time since first or last use. The effect seemed to last for more than 20 years after stopping taking the drugs. However, when duration of HC use was taken into account, the reduced risks of epithelial ovarian neoplasms according to age at first use, time since first use or time since last use were no longer statistically significant.

We were not able to carry out any meaningful detailed analysis according to specific HC brands due to small numbers of exposed ovarian cancer cases. None of the tests for heterogeneity across subgroups of women with invasive and borderline tumours (classified according to ever/current/never use of HC, use of COCs and POPs, and duration of use of HC, COCs and POPs) were statistically significant (data not shown).

## DISCUSSION

In our study, we confirm a strong inverse risk association between use of HC and epithelial ovarian neoplasms. Women who used HC for many years were at particularly lower risk as compared to women who were never users. We found no evidence of differential effect of HC use on the development of invasive or borderline tumours. Nor was the impact of use of combined estrogen–progestin contraceptives and progestins-only contraceptives different from each other with regard to cancer risk.

The strength of this study is the prospective design, the large size, and the complete follow-up based on linkage to national registers (Lunde *et al*, 1980). Since cancer registration is compulsory in both countries, the assessment of cases is virtually complete. A limitation of this study is the lacked of systematic validation of the self-administrated questionnaire. Studies of the reproducibility and validity of questions about HC use have been carried out by others (Coulter *et al*, 1986; Nischan *et al*, 1993; Hunter *et al*, 1997). Although accurate reporting of HC use is important, HC use in both case–control and cohort studies is obtained retrospectively and distant use might be poorly recalled.

Since the information about HC was obtained at enrolment, changes in exposure status are not accounted for. Only 9% of the women were current users of HC at enrolment. Particularly the youngest women in the cohort might have changed the brand of HC used, ceased or started with HC during the follow-up period. Similarly, women aged 40 years or more at enrolment, who were current HC users, had most probably ceased their use during the follow-up. Such changes in the patterns of use during follow-up may have caused misclassification of exposure. Many important reproductive factors for ovarian cancer are likely to vary in ways that are related to each other (e.g. women who have had children may then start contraceptives, women who stop contraceptives may then get pregnant, etc.), and may be misclassified over time in our study. The impact of such possible misclassification will only be known once we have updated information on the entire cohort, which is ongoing at the moment. However, due to their ages, we believe that most participating women had probably ended their reproductive life at enrolment, so that changes in parity during follow-up probably have a very modest impact as a confounder on the overall association with HC. It is expected that at least a few women would have undergone hysterectomy and/or tubal ligation during follow-up, but they should not change substantially the association between use of HC and ovarian cancer risk.

Our study indicates that the positive effect of HC on ovarian neoplasms is associated with the duration of use, the effect rising with each additional year of use. However, the finding of an important reduced risk in women with very long duration (15 or more years) was based on only one case. After including the duration in the analytical models, no independent effect was observed for age at first use, or for time since the first or last use. Thus, duration, rather than time of use, seems to be the most important in determining a decreasing ovarian cancer risk.

Few analyses of prospective studies on HC and ovarian neoplasms are available and three out of the four studies were based on small numbers (Ramcharan *et al*, 1980; Beral *et al*, 1988; Vessey and Painter, 1995). Only the most recent, the Nurses' Health Study (Hankinson *et al*, 1995), did not confirm a protective effect (including both *invasive and borderline cases*). In the analysis of the Nurses' Health Study, based on 260 cases, the exposure was restricted to shorter durations of use than in other studies. The nurses were aged 30–55 years in 1976 at enrolment, while the women in our study were 30–49 years in 1991. Thus, the opportunity to be ever exposed to HC differed greatly.

In our study, there was no statistically significant difference between the effect of use of HC on risk of invasive or borderline neoplasms, in agreement with most previous studies (Riman, 2003).

Epidemiological evidence from case–control studies on use of COCs and ovarian cancer risk is well defined and consistent (IARC Working Group, 1999): at least 20 out of 21 studies published between 1980 and 1997 found RRs below unity. Little information is available on progestins-only contraceptives and risk, but none of the published studies showed any alteration in risk according to the type of hormonal content (IARC Working Group, 1999). Ovarian cancer incidence rates have remained constant or have declined in Norway and Sweden over the last two decades (Adami *et al*, 1990; Bjorge *et al*, 1997). At the same time, both the incidence and the duration of HC use in the Nordic countries have been increasing. Since the incidence of ovarian cancer is already appreciable in middle age, and the prognostic outlook is gloomy, a reduction of risk attributable to HC use would be important in any risk–benefit evaluation (Parazzini *et al*, 2000). Further research would be necessary to establish whether there has been a direct link between rising HC use and declining ovarian cancer incidence in Norway and Sweden.

### Contributions

M Kumle and E Weiderpass were responsible for data management in Norway and Sweden, respectively, and for co-ordination of manuscript writing. T Braaten performed the combined Norwegian–Swedish data management and performed the statistical analysis of the data. E Lund and H-O Adami, the principal investigators and initiators of the project in Norway and Sweden, respectively, participated in all phases of the study.
